# The clinico-radiological paradox of cognitive function and MRI burden of white matter lesions in people with multiple sclerosis: A systematic review and meta-analysis

**DOI:** 10.1371/journal.pone.0177727

**Published:** 2017-05-15

**Authors:** Daisy Mollison, Robin Sellar, Mark Bastin, Denis Mollison, Siddharthan Chandran, Joanna Wardlaw, Peter Connick

**Affiliations:** 1 Centre for Clinical Brain Sciences, University of Edinburgh, Edinburgh, United Kingdom; 2 Department of Actuarial Mathematics and Statistics, Heriot-Watt University, Edinburgh, United Kingdom; Heinrich-Heine-Universitat Dusseldorf, GERMANY

## Abstract

**Background:**

Moderate correlation exists between the imaging quantification of brain white matter lesions and cognitive performance in people with multiple sclerosis (MS). This may reflect the greater importance of other features, including subvisible pathology, or methodological limitations of the primary literature.

**Objectives:**

To summarise the cognitive clinico-radiological paradox and explore the potential methodological factors that could influence the assessment of this relationship.

**Methods:**

Systematic review and meta-analysis of primary research relating cognitive function to white matter lesion burden.

**Results:**

Fifty papers met eligibility criteria for review, and meta-analysis of overall results was possible in thirty-two (2050 participants). Aggregate correlation between cognition and T2 lesion burden was *r* = -0.30 (95% confidence interval: -0.34, -0.26). Wide methodological variability was seen, particularly related to key factors in the cognitive data capture and image analysis techniques.

**Conclusions:**

Resolving the persistent clinico-radiological paradox will likely require simultaneous evaluation of multiple components of the complex pathology using optimum measurement techniques for both cognitive and MRI feature quantification. We recommend a consensus initiative to support common standards for image analysis in MS, enabling benchmarking while also supporting ongoing innovation.

## Introduction

Cognitive impairment is seen in 43–70% of people with multiple sclerosis (MS), exhibiting a variable pattern of deficits between individuals [1]. The most frequently detected deficits include a reduction in information processing speed, executive functions, attention, and long-term memory. Impairment of information processing may represent the core cognitive deficit [2], consistent with the model of a disconnection syndrome [3]. The underlying pathology is complex, including both focal and diffuse abnormalities of the central nervous system that affect both white and grey matter structures [4]. Components of this pathology have been increasingly amenable to in vivo quantification through magnetic resonance imaging (MRI) and associated image analysis techniques [5]. The impact of pathology on phenotype is also influenced by lifetime intellectual enrichment (‘cognitive reserve’) [6], lifestyle variability (cognitive leisure), as well as comorbidities, ageing, and medications.

Although aspects of the ‘global’ pathological burden affecting the brains of people with MS can be readily estimated by abnormalities such as T2 hyperintense lesions that are visible on structural MRI, limited correlation exists between these measures and the clinical phenotype. This has been termed the ‘clinico-radiological paradox’ (CRP) and is well described for both physical and cognitive impairments [7]. The CRP presents a fundamental challenge with respect to mechanistic understanding of the relationship between pathology and phenotype in MS, and to the use of MRI metrics in clinical decision-making at the individual-subject level. Several explanations have therefore been proposed in order to resolve the CRP, including recognition that summation of whole-brain metrics fails to account for variability between subjects in the spatial patterning of multifocal pathology [8]. Although such consideration has a clear corollary through the fundamental principles of localisation in clinical neurology, it provides a less satisfactory explanation with respect to cognitive impairments, particularly for cognitive functions such as information processing speed where the functional neuroanatomy involves widespread connectivity between brain regions [9, 10].

Additional potential contributors to the ‘cognitive CRP’ include fundamental issues with the evaluation of cognition such as whether any existing test can isolate and quantify a neuroanatomically distributed cognitive function. Or, whether multidimensional cognitive assessment through ‘cognitive batteries’ provides a valid quantitative assessment of ‘global’ cognitive performance. Such ‘global’ evaluation may be contingent on pre-requisite and relatively localised functions such as sustained attention that are subject to the potential confound of variable spatial patterning. Although mitigated by the use of batteries with normative data simultaneously developed for component tests, judgement with respect to the latter remains a fundamental principle applied in clinical neuropsychology [11]. Separately, critical issues also arise in the quantification of total pathological burden by structural brain MR imaging. These include the known insensitivity of existing metrics for potentially critical aspects of disease pathology such as grey matter lesions [12] and a failure to adequately quantify neuroaxonal loss or underlying subvisible/diffuse pathology. Critically, these aspects of the complex pathology may be independent at the individual-subject level [13], rendering assessment of T2 hyperintense lesion burden as an inadequate account of the total (multifaceted) pathological burden.

In addition, modest correlation of total MRI-visible white matter lesion burden to cognitive status may reflect attenuation due to psychometric limitations in the methodologies for quantification of both cognitive and MRI features [14], as well as aspects of study design including participant selection. We therefore performed a systematic review and meta-analysis of the published literature describing the relationship between cognitive function and the total burden of white matter pathology detected by standard structural brain MRI. Our aim was first to confirm the modest correlations that have been previously described [15], and second to explore the potential methodological issues that may affect the observed relationship.

## Methods

Design of the systematic review, meta-analysis, and manuscript was based on PRISMA (‘Preferred Reporting Items for Systematic Reviews and Meta-Analyses’) guidelines [16].

### Protocol, information sources and search strategy

The study protocol was documented in advance. Medline, Embase, and Web of Science databases were searched for English language papers on 1st July 2015, with no date restrictions (S1 Appendix). Review articles were excluded, but relevant reviews published in the last 10 years were screened for references. Archives of the journals Neurology, Multiple Sclerosis and the American Journal of Neuroradiology were also hand-searched for relevant articles published in the previous ten years. Search terms were: ‘magnetic resonance imaging’, ‘multiple sclerosis’, ‘cognitive’, ‘cognition’, related terms and abbreviations of these.

### Study selection and eligibility criteria

Initial screening of abstracts was performed by a single author (DM). Full articles were then retrieved and eligibility assessment performed in a standardized manner, with a final decision over study inclusion taken in consensus with a second reviewer (PC). Eligibility criteria were: English language and peer-reviewed publications reporting data from adults with clinically definite MS as primary research with a primary aim of relating cognition to T1w, T2w, FLAIR or PD metrics of total brain white matter lesion burden. Imaging outcomes for total lesion volume or area, and lesion counts or scores, were all accepted as valid measures of whole brain lesion burden. Similarly, any measure of cognitive function with face-validity was accepted. Studies were excluded if reporting exploratory or secondary analysis, or if lesion burden was only related to longitudinal change in cognitive function. Where studies examined both cross-sectional and longitudinal outcomes, the baseline cross-sectional analyses were used. When overlap of reported cohorts was identified and clarification from the original investigators was not possible, a conservative approach was adopted with inclusion of only the earliest dated relevant article. Studies within the systematic review were suitable for meta-analysis if they reported an overall effect for the relationship of imaging metrics to a single measure of cognition defined by either a single cognitive test, or a summary result from a cognitive battery.

### Data collection

Data was extracted by a single author (DM) using a standardized form that captured (1) characteristics of the participants, including age, sex and disease phenotype; (2) cognitive testing methods including blinding and identity of the tester; (3) image acquisition methods; (4) image analysis methods including training and blinding of investigators, software tools used, whether measures of intra- and inter-rater reliability were provided; and (5) statistical analysis methods including controlling for potential confounding factors. A study quality assessment tool (S2 Appendix) was also developed based on STROBE (‘Strengthening the Reporting of Observational studies in Epidemiology’) guidelines [17] to evaluate the risk of bias in individual studies. The authors for one paper were contacted for further information and numerical data was provided.

### Summary measures and synthesis of results

Summary measures were recorded if relating MRI metrics to an overall measure of cognitive function or to a single cognitive test. Where summary measures were provided both unadjusted and adjusted for potentially confounding clinical covariates, adjusted results were used. Correlation coefficients or the difference in lesion burden between groups defined by cognitive status were accepted as summary measures, with preference given to correlations if both were available [18]. All reported summary measures were converted into effect sizes and inverted as necessary so that negative values always indicated an association of lower cognitive scores to higher lesion burdens. Standardized mean differences were calculated from studies reporting group comparisons, prior to conversion to equivalent correlations (*r*). An approximation to the standard deviation was estimated as necessary based on available measures of dispersion (e.g. interquartile range or range). In studies with two impaired groups defined by specific cognitive deficits, these groups were combined before calculation of a standardized difference from a non-impaired group. The Fisher’s *z* transformation was used prior to calculation of an aggregate summary effect, with conversion back to *r* for reporting of overall meta-analysis findings and confidence intervals.

An aggregate summary effect was calculated using maximum likelihood estimation [19], taking into account the size of the various studies; this method allows incorporation of those studies reporting non-significant results without providing their estimate. Separate analyses were carried out for studies measuring hyperintense lesion burden on T2w, FLAIR and/or PD sequences, and for the subgroup of studies evaluating T1w hypointense lesion volume. Heterogeneity was assessed by Cochran’s Q and the I^2^ statistic [20], based on the studies providing specific estimates of the effect size. All analyses were performed using the statistical software *R*, version 3.2.4.

### Risk of bias across studies

Our eligibility criteria required a stated primary aim to evaluate the relationship between cognitive status and brain imaging metrics so that we might minimize the influence of reporting bias from *post hoc* analyses. Within the included studies, we recorded analyses that were described without results being provided. A funnel plot was also evaluated visually and tested formally using Egger’s regression test for asymmetry.

### Additional analyses

An alternative aggregate effect size was calculated using quality scores as an additional scaling factor. A sensitivity analysis examining the effect of using an alternative random-effects model, with DerSimonian and Laird methodology, was carried out for all studies providing data compatible with precise estimates of the effect size. Subgroup analyses of studies using the Paced Auditory Serial Addition Test (PASAT) and Symbol Digit Modalities Test (SDMT) were also pre-specified to investigate whether focusing on distributed cognitive function would improve correlations with overall lesion burden and replicate previous findings [15].

## Results

### Study selection

**Fig 1:**
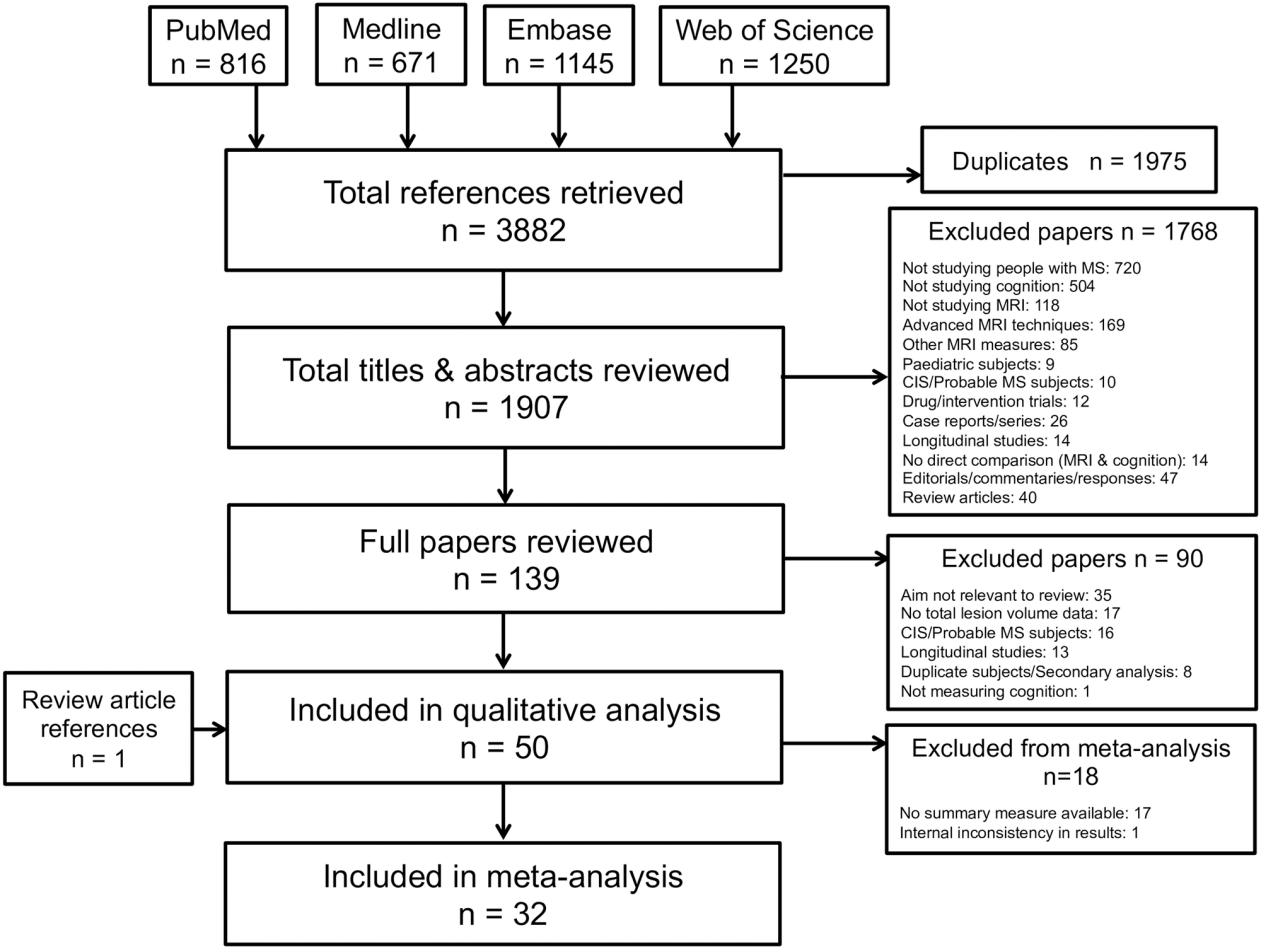
Flowchart showing articles retrieved and considered at each stage of the review process.

A total of 3882 studies were identified by the initial literature search, 1975 of which were duplicates (Fig 1). Year-on-year increases were seen in the publication rate identified through the initial search (S1 Fig). No additional studies were included following hand searching of journal archives. After review of abstracts, 139 manuscripts were retrieved. Ninety were subsequently excluded, most frequently (35/90 = 39%) because the study aim was not relevant. A total of fifty papers met all inclusion criteria [21–70] spanning the period 1987–2015.

Thirty studies provided usable summary measures relating hyperintense T2w/FLAIR/PD lesion burden to cognitive function. Two studies reported a ‘non-significant’ result and one study was excluded from meta-analysis as the reported summary measure was internally inconsistent with other reported results and significance levels. The remaining seventeen studies did not provide results suitable for use in meta-analysis, reporting only individual results for each cognitive subtest (n = 12) or multiple regression modeling with simultaneous assessment of several brain imaging metrics (n = 5). Thirteen studies related cognition to T1 hypointense lesion burden, of which eleven provided usable summary measures and two reported ‘non-significant’ results.

### Participant characteristics

**Fig 2:**
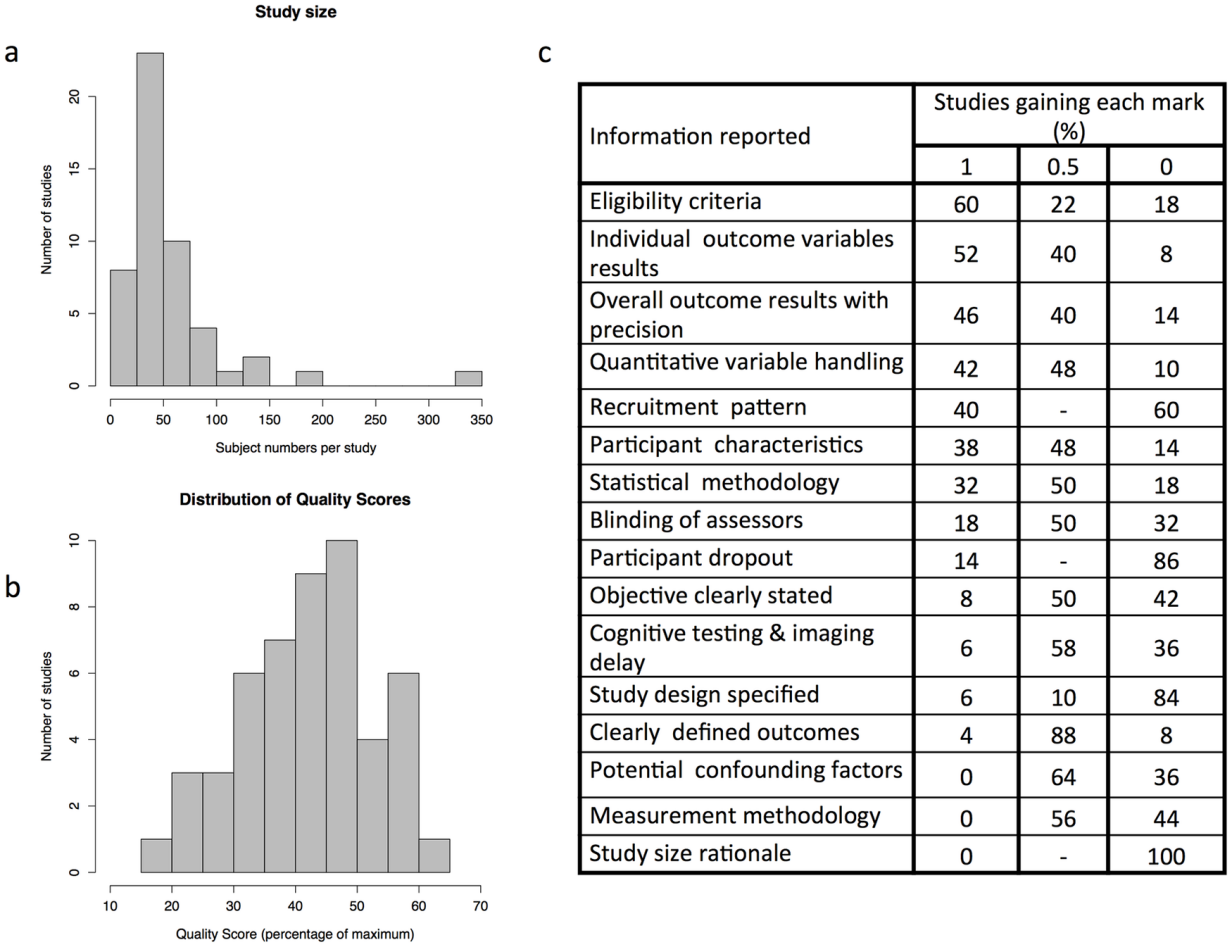
Showing factors relevant to study quality including histograms of a) numbers of participants with MS in individual studies and b) overall quality scores, and c) the reporting of individual factors contributing to the overall quality score.

The total number of subjects from all included studies was 2891. Individual study size ranged from 17 to 327 participants (mean 58, median 45; Fig 2a). Forty-four studies specified the sex ratio, all but one having a female majority. The range of mean participant age (provided in 47/50 studies) was 31–55 years. No study used age of disease onset in its eligibility criteria. Twenty-six studies included participants with a mixture of disease courses; thirteen studies recruited exclusively relapsing-remitting disease, six studies progressive disease, two ‘benign’, and three did not specify the participants’ disease course.

### MR brain imaging acquisition

The majority (29/50 studies) used 1.5T scanners. Ten studies used scanners with below 1.5T magnets for some or all participants’ imaging, seven used 3T scanners, one used both 1.5 and 3T scanners and three did not specify the scanner field strength. Details of the imaging protocol were given in all but seven studies.

### Image analysis

The sequence(s) used to measure lesion volume was specified in forty-three studies. Twenty-six specified the number of people involved in the lesion analysis, a single observer in fourteen. The anatomical boundaries of evaluation were explicitly defined in two studies and a sample image was provided by five studies. A wide variety of approaches were used for the quantification of lesion burden. These included lesion counts (two studies) or weighted lesion scores (six studies), manual lesion outlining either on hard copies (two studies) or within viewing software (six studies), and the use of semi-automated methods (thirty-one studies). Of the six studies using lesion scores, five different scoring systems were used. One study used both manual and semi-automated measurements (for different sequences), one used manual lesion outlining and an absolute lesion count, and in one study the methodology was unclear. In the thirty-two studies using semi-automated measurement tools, the software used was specified or references provided in 25 studies (78%), covering fourteen different software packages. In eighteen of these studies the named software was publically available (eleven different softwares). The remaining studies did not specify their software. A manual editing stage for software-generated lesion masks was specified in five studies (16%) and the person performing this was described in two studies. In the ten studies using fully manual lesion outlining, the person performing this was described in six. Only two studies provided an indication of inter-observer agreement and one study intra-observer reproducibility. Seven studies mentioned previous measures of reproducibility or results on training data sets. Only five percent of studies calculating a lesion volume or area (2/42) normalized to intracranial volume.

### Cognitive testing

The cognitive assessor and their training was unclear in thirty-eight studies. Of defined batteries, the most commonly used was Rao’s Brief Repeatable Battery (12/50), followed by the Minimal Assessment of Cognitive Function in MS (5/50), used with modifications or additional tests in eight (67%) and two (40%) studies respectively. Unique collections of tests were found in twenty-seven studies. The SDMT or PASAT were used either exclusively or as part of a wider battery in thirty studies. Substantial variability was seen in how raw cognitive scores were processed prior to their use in the evaluation of a possible relationship with imaging metrics. Methods included use of unadjusted scores, standardization, and the deployment of group classifiers. Standardization was performed using either historic- (published or unpublished) or contemporary- (matched or unmatched for participant characteristics) control data. Group classifiers were either based on internal (patient) or external (normative) reference cohorts. The specific thresholds used to define impairment on individual tests were also variable, including 1, 1.5, and 2 standard deviations from the reference mean, and those based on centiles. Moreover, the number of failed tests used to define overall cognitive impairment was also variable (S1 Table). Consideration of the effect of potential confounders also varied between studies, both in the recording of relevant data and whether it was adjusted for in the analysis. Some studies adjusted for age (n = 18), sex (n = 12), education level (n = 13) and/or affective disorders (n = 15). Drug treatments and premorbid IQ were both adjusted for in three studies. Cognitive leisure activities were neither measured nor adjusted for in any study.

### Statistical analysis

Summary measures were provided through univariate correlations (n = 37) and/ or group comparisons based on cognitive status (n = 24). Four studies divided participants into groups dependent on radiological features. Fourteen studies constructed statistical models predicting cognitive performance based on imaging and other laboratory, demographic, or clinical markers.

### Reporting quality and risk of bias within studies

A range of study-specific quality scores was seen (mean 42%, SD 11%; Fig 2b). Among individual elements of the composite quality score, complete reporting was provided most frequently for eligibility criteria and outcome measures (Fig 2c). In contrast, no study provided complete reporting of potential confounding factors, measurement methodology, or study size rationale.

### Results of individual studies

Studies directly reporting correlation coefficients relating cognitive performance to T2 hyperintense lesion burden ranged from -0.6 to -0.23. Standardised mean differences ranged from -2.70 to 0.23, equivalent to correlations of -0.80 to 0.11.

### Synthesis of results

**Fig 3:**
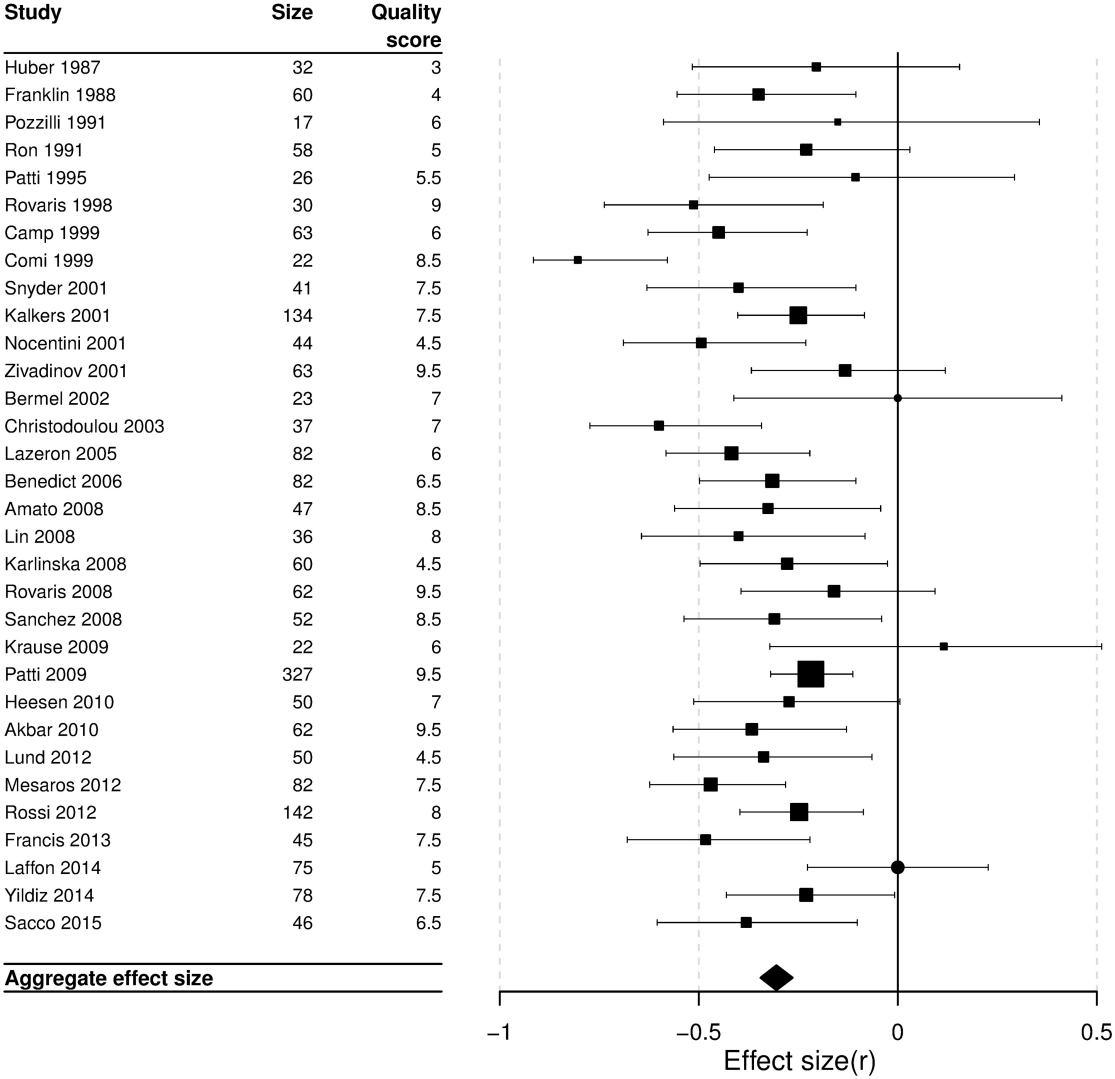
Forest plot of the individual studies showing their effect sizes as correlation coefficients. Box sizes are inversely proportional to study variance. Manuscripts reporting “non-significant” results without a point estimate are represented by circles. Aggregate effect size: r = -0.30; 95% confidence interval: -0.34, -0.26.

The aggregate effect size relating cognitive performance to T2 hyperintense lesion burden was *r* = -0.30 (95% confidence interval: -0.34 to -0.26; Fig 3). There was evidence of possible heterogeneity (Q = 43.62, df = 29, p = 0.04; *I*^*2*^ = 33.5%). The aggregate effect size relating cognitive performance to T1 hyperintense lesion burden was *r* = -0.26 (95% CI: -0.32, -0.20; Q = 20.4, df = 10, p = 0.025, *I*^*2*^ = 51.0%, see S3 Appendix for further details).

### Risk of bias across studies

Funnel plot inspection (S2 Fig) and Egger’s test of asymmetry gave an equivocal result (p = 0.05). We therefore explored possible underlying sources of heterogeneity [71]. Reporting biases could not be evaluated as study protocols were not published prospectively. Despite methodological heterogeneity apparent from our quality scoring, no correlation was seen between overall quality score and effect size (r = -0.18, p = 0.34). Exploratory meta-analysis using quality scores as an additional weighting factor returned an effect size similar to that of our primary analysis (*r* = -0.30; 95% CI: -0.36, -0.24). In order to explore the possibility of ‘true heterogeneity’ between study effect sizes, we performed a sensitivity meta-analysis using a random effects model, giving an overall effect size similar to that of our primary analysis (*r* = -0.33; 95% CI: -0.38, -0.27). Further sensitivity analyses, comparing scanner field strength and type of lesion quantification method did not demonstrate a measurable subgroup difference in heterogeneity from the small number of studies using high (3T) or low (below 1T) field scanners, or from those using lesion counts or scores.

### Additional analyses

#### Alternative cognitive endpoints

Exploratory meta-analyses were performed on two widely used measures of information processing speed (IPS), the SDMT and PASAT (S4 & S5 Appendices). Our *a priori* hypothesis was that total lesion burden would have a stronger correlation with these tests of distributed cognition function (IPS) compared to the mixture of distributed and localized functions in our primary analysis. The summary effect size for SDMT was *r* = - 0.37 (95% CI: -0.43, -0.31; n = 13 studies) and for PASAT was *r* = - 0.28 (95% CI: -0.34, -0.22; n = 15 studies).

## Discussion

Our results confirm a modest correlation (*r = -* 0.30) between MRI measures of total brain white matter lesions and cognitive function in people with MS. Although some variability was observed between studies in the magnitude of the reported relationship, no large (>100 participants) single study demonstrated a strong correlation. We therefore sought to explore whether technical and methodological factors may have been important in attenuating the reported correlation.

Substantial variability was seen with respect to study design, including the approaches used to quantify both cognitive function (see review by Fischer *et al* [72]) and lesion burden, and the adjustment for other variables that influence cognition (e.g. education, premorbid IQ and drugs). For cognitive assessment, this may represent a largely historic issue as a global movement is now established to harmonise evaluation and scoring through the Brief International Cognitive Assessment for MS (BICAMS) initiative [73]. In contrast, the optimum method to generate quantifiable measures of lesion burden from brain imaging data lacks emergent consensus. Recent initiatives to harmonise MR acquisition protocols are welcome [74, 75], however no similar initiative exists for image analysis techniques. Semi-automated approaches were the most frequently used (62%) and therefore merit particular consideration. While effective manual editing is clearly dependent on adequate training of the operator, the automated (software) component is more challenging to benchmark. We recommend that authors should routinely report the software used. Separately, the field risks delaying progress and reducing the potential for collaboration due to the many differing software packages used. Of the twenty-four studies naming software, ten different publicly available (commercial or open source) packages were used, and a further three packages that were developed ‘in house’. To our knowledge, no comparative study has been performed on a common dataset to evaluate agreement between these varied approaches. It is therefore our view that a new consensus initiative is required to support an image analysis framework in MS that enables benchmarking while also supporting ongoing innovation.

Despite our finding of substantial methodological variability between studies, formal testing for heterogeneity in our primary meta-analysis returned an equivocal result. This indicates that methodological variability between studies cannot provide a sufficient explanation for the cognitive-CRP. However, measurement errors within all published studies may have attenuated observed correlations in the face of a higher ‘true’ correlation [14]. Greater recognition and transparency around measurement error for both cognitive and lesion-burden quantification would therefore be beneficial to the field.

As previously noted, resolving the cognitive-CRP may require consideration of the spatial patterning of lesions with simultaneous evaluation of other aspects of MS pathology that may be both phenotypically relevant and independent from the burden of white matter hyperintensities [76, 77]. However, a further potential contributor is the pathological variability of white matter T2 hyperintense lesions. Conventional MRI is unable to distinguish the extent of intra-lesional inflammatory infiltrate, demyelination, remyelination, axonal damage, or gliosis [78]. If cognitive impairment reflects only some of these pathological features, then the remainder will contribute only measurement error; improved MR-based quantification of individual lesion characteristics may therefore be critical.

Our findings may have been limited by an overly inclusive approach to both the evaluation of cognition and white matter lesion burden. With respect to the former, we saw a higher aggregate correlation between white matter lesion burden and cognition measured by the SDMT–a measure of information processing speed, understood to reflect widely distributed brain connectivity–than was seen for cognition as defined in the primary analysis. Notably, relatively few studies in our review used >1.5T field-strength scanners, in part reflecting the recent shift away from exploring the relationship between phenotype and T2 hyperintense lesion burden, focusing instead on the possible relevance of other MR metrics. We therefore interpret our sensitivity analysis for the effect of magnet field strength to lack sufficient data for a definitive conclusion. We would encourage re-evaluation of this relationship as the literature evolves with respect to 3T acquisition and/or in cohorts scanned on both low and high magnetic field scanners. Finally, a substantial body of potentially relevant data was excluded from our review as the primary aim of the study was unclear or reported findings were secondary/exploratory analyses. Finally, despite our best efforts to apply a systematic approach, all reviews are conducted by researchers who bring unconscious bias [79].

## Conclusions

We replicate the finding that modest correlation (*r = -* 0.30) exists between MRI measures of total brain white matter lesion burden and cognitive function in people with MS. However, the quantification techniques for both cognitive and MR features were highly variable, and this may have attenuated the observed strength of the association. An accurate assessment of the relationship requires optimum measurement techniques; this is a prerequisite to meaningful investigation of the clinico-radiological paradox through simultaneous evaluation of multiple components of the complex pathology. We therefore make the following recommendations:

1. A new consensus initiative is advanced to support an image analysis framework in MS that enables benchmarking while also supporting ongoing innovation.
2. Greater recognition and transparency are fostered around measurement error for both cognitive and lesion burden quantification.
3. Reporting of observational research adheres to best-practice guidance such as provided by the STROBE statement.
4. Attempts to resolve the cognitive clinico-radiological paradox should adopt a more multidimensional approach to understanding white matter lesions with simultaneous consideration to multiple elements of the quantifiable pathology, whilst also incorporating potential clinical confounders of the relationship.

## Supporting information

S1 Appendix Record of database search. (DOCX)

S2 Appendix Quality assessment tool for evaluation of manuscripts, based on the STROBE checklist. (DOCX)

S3 Appendix Sub-analysis of studies relating T1 hypointense lesion volume to overall cognitive performance. (DOCX)

S4 Appendix Sub-analysis of studies relating T2 hyperintense lesion volume to Symbol Digit Modalities Test (SDMT) performance. (DOCX)

S5 Appendix Sub-analysis of studies relating T2 hyperintense lesion volume to Paced Auditory Serial Additions Test (PASAT) performance. (DOCX)

S6 Appendix Database containing relevant data extracted from primary literature. (XLSX)

S7 Appendix PRISMA checklist. (DOC)

S8 Appendix PRISMA flow diagram. (DOC)

S1 Fig Number of results retrieved from database search by year of publication. The 2015 point is an extrapolated value from the 6-month figure.
(EPS)

S2 Fig Funnel plot of effect sizes, on Fisher’s z scale, against the inverse of their standard error (itself inversely related to study size) with asymmetry towards increased reporting of stronger correlations for smaller study sizes. The vertical dashed line indicates the summary effect on the same scale (z = -0.32).
(EPS)

S1 Table Summarising cognitive testing and scoring protocols. (DOCX)
