# Supplementary material for: The clinico-radiological paradox of cognitive function and MRI burden of white matter lesions in people with multiple sclerosis: A systematic review and meta-analysis
Source: PLoS One. 2017 May 15;12(5):e0177727. doi: 10.1371/journal.pone.0177727 (PMC5432109; doi:10.1371/journal.pone.0177727)
Supplement: S1 Appendix — (DOCX) [file pone.0177727.s001.docx]

**S1 Appendix:** Record of database search

**1. Medline (searched via OVID platform) 01/07/15**

Using search strategy:

| **#** | **Searches** | **Results** |
| --- | --- | --- |
| 1 | multiple sclerosis.mp. or Multiple Sclerosis/ | 56333 |
| 2 | magnetic resonance imaging.mp. or Magnetic Resonance Imaging/ | 353245 |
| 3 | mri.mp. | 144054 |
| 4 | mr imaging.mp. | 32694 |
| 5 | 2 or 3 or 4 | 382327 |
| 6 | cognitive.mp. or Cognitive Reserve/ or Delirium, Dementia, Amnestic, Cognitive Disorders/ or Cognitive Science/ or Mild Cognitive Impairment/ | 208670 |
| 7 | Cognition Disorders/ or Cognition/ or cognition.mp. | 133903 |
| 8 | 6 or 7 | 260390 |
| 9 | 1 and 5 and 8 | 742 |
| 10 | limit 9 to english language | 672 |
| 11 | limit 10 to retracted publication | 1 |
| 12 | 10 not 11 | 671 |

**2. Embase (searched via OVID platform) 01/07/15**

| **#** | **Searches** | **Results** |
| --- | --- | --- |
| 1 | multiple sclerosis.mp. or multiple sclerosis/ | 94203 |
| 2 | magnetic resonance imaging.mp. or nuclear magnetic resonance imaging/ | 588177 |
| 3 | cognition/ or cogniti*.mp. | 426368 |
| 4 | 1 and 2 and 3 | 1844 |
| 5 | limit 4 to english language | 1755 |
| 6 | limit 5 to (conference abstract or conference paper or conference proceeding or “conference review”) | 610 |
| 7 | 5 not 6 | 1145 |

**3. Web of Science 01/07/15**

| **#** | **Searches** | **Results** |
| --- | --- | --- |
| 1 | TOPIC: (magnetic resonance imaging) OR TOPIC: (mri) OR TOPIC: (mr imaging) | 343471 |
| 2 | TOPIC: (cogniti*) | 426125 |
| 3 | TOPIC: (multiple sclerosis) | 90559 |
| 4 | #3 AND #2 AND #1 | 1396 |
| 5 | #3 AND #2 AND #1  Refined by: DOCUMENT TYPES: (ARTICLE OR EDITORIAL MATERIAL OR REVIEW) | 1324 |
| 6 | #3 AND #2 AND #1  Refined by: DOCUMENT TYPES: (ARTICLE OR EDITORIAL MATERIAL OR REVIEW) AND LANGUAGES: (ENGLISH) | 1250 |

**4. PubMed 01/07/15**

To ensure recent papers not yet indexed on Medline were also included, the PubMed database was searched using the following search strategy:

***((((((magnetic resonance imaging) OR MRI)) OR MR imaging)) AND ((cognition) OR cognitive)) AND multiple sclerosis AND (English[lang])***

→ 816 results retrieved
