# Supplementary material for: The clinico-radiological paradox of cognitive function and MRI burden of white matter lesions in people with multiple sclerosis: A systematic review and meta-analysis
Source: PLoS One. 2017 May 15;12(5):e0177727. doi: 10.1371/journal.pone.0177727 (PMC5432109; doi:10.1371/journal.pone.0177727)
Supplement: S2 Appendix — (DOCX) [file pone.0177727.s002.docx]

**S2 Appendix: Quality assessment tool for evaluation of manuscripts, based on the STROBE checklist**

**Instructions:** Where all key points are met, 1 point is awarded. Where the study meets most but not all of the applicable criteria, or only part of the relevant information is provided, a score of 0.5 is awarded.

|  | **Criterion** |  | **Score [0]/[0.5]/[1]** |
| --- | --- | --- | --- |
|  | INTRODUCTION | | |
| **1** | **OBJECTIVE: State specific objectives, including any prespecified hypotheses.**   - The study should have a clearly stated objective mentioning white matter lesion volume as a metric of interest (awarded 0.5). - Full credit will only be given where the objective specifies what imaging sequence(s) will be used to measured lesion volume and what cognitive measure is used to examine the relationship between the two outcomes. |  |  |
|  | METHODS | | |
| **2** | **STUDY DESIGN: Present key elements of study design early in the paper.**   - The study design should be presented clearly, i.e. retrospective or prospective recruitment, case-control studies, or a sub-study of part of a larger study. - Prospective recruitment to address the study objective is considered preferable and a clear statement of this is needed for 1 point. A retrospective study design will be awarded 0.5. - Where participants are taken from a cohort being used for multiple (sub)studies, a maximum of 0.5 can be awarded, unless cognition and imaging relationships are clearly the primary aim of the overall study and cross-sectional baseline data are being used. Enough detail should be provided to ensure results are not duplications of other published work. |  |  |
| **3** | **SETTING: Describe the setting, locations, and relevant dates, including periods of recruitment, exposure, follow-up, and data collection.**   - The dates of recruitment and testing should be provided. - The delay between cognitive testing and imaging should be specified and less than 6 months. - Both the above criteria are necessary for 1 point, either alone will be awarded 0.5. - A description of the clinical setting (e.g. tertiary referral centre, multiple district general hospitals etc.) is considered optimal, but is not necessary for full credit. |  |  |
| **4** | **PARTICIPANTS: Give the eligibility criteria, and the sources and methods of selection of participants.**   - The authors should have clearly stipulated the criteria they used to include (and if applicable, to exclude) subjects into the study. A positive statement of who was sought for recruitment (whether any person with MS, or e.g. only people with a particular clinical phenotype) with relevant exclusion criteria is necessary for 1 mark. - Participants should not be excluded solely on the basis of higher levels of physical or cognitive disability, and where recruited subjects were unable to tolerate MRI this should be recorded. |  |  |
| **5** | **RECRUITMENT:**   - The recruitment should be either a consecutive or random sample of eligible participants. Where this is unclear, the study will be awarded 0. |  |  |
| **6** | **VARIABLES: Clearly define all outcomes, exposures, predictors, potential confounders, and effect modifiers. Give diagnostic criteria, if applicable.**   - The cognitive tests performed should be specified. Whether results were interpreted relative to a control population or published norms should be clearly stated/described. - The definition of total lesion volume should be clearly defined, including the brain regions covered (whether deep grey matter included or excluded, whether posterior fossa included and how defined), the MRI sequence used for measurement and whether the results were adjusted for total (estimated) brain volume. - Clear definitions as above are required for both imaging and cognitive outcomes for a score of 1. Where one of these is unclear, a maximum of 0.5 will be awarded. |  |  |
| **7** | - Potential confounding factors, including age, sex, education, drugs, pre-morbid IQ, pre-morbid cognitive leisure activities & affective disorders, should be measured. A score of 1 will be awarded where all these are identified, and 0.5 if ≥4 of them. |  |  |
| **8** | **DATA SOURCES/MEASUREMENT: For each variable of interest, give sources of data and details of methods of assessment (measurement). Describe comparability of assessment methods if there is more than one group.**   - The person(s) performing the cognitive testing should be identified, with their level of training/experience. - Enough data should be provided to replicate the imaging sequences. This should included at least the type of sequence performed (e.g. spin echo, gradient echo), slice thickness and inter-slice interval, and preferably the pulse parameters (TE, TR, flip angle, FOV, matrix size), number of slices and magnet strength. - The method for measuring/estimating lesion volume should be clearly described, with details of the software package used if applicable. The person(s) performing the analysis should be identified with their level of training/experience. Measures of intra-/inter-observer variability should be provided. - All of the above criteria must be met for a score of 1; where ≥50%, but not all, of the relevant information is presented, the study will be awarded 0.5. |  |  |
| **9** | **BIAS: Describe any efforts to address potential sources of bias**   - Cognitive testing and image analysis should both be performed by individuals blind to the results of the other and this should be clearly stated. Where there is only a statement that the image analysis was performed blind to the cognitive results, 0.5 will be awarded, otherwise the study will be scored 0. - Ideally the image analysis and cognitive testing should be carried out blind to (as far as possible in the case of cognitive testing) all data on clinical status and confounding factors. |  |  |
| **10** | **STUDY SIZE: Explain how the study size was arrived at.**   - A calculation of study size should be provided. |  |  |
| **11** | **QUANTITATIVE VARIABLES: Explain how quantitative variables were handled in the analyses. If applicable, describe which groupings were chosen and why.**   - Ideally, the full range of cognitive scores and lesion volumes will be used for the analysis, with or without transformation to Z-scores. This should be clearly stated and correlations using the full range of values or correlations by rank will be awarded 1 point - If participants are categorised into groups by results of cognitive status (or, less likely, lesion volumes) the justification of the group definitions should be provided and boundaries pre-specified. A maximum of 0.5 will be awarded where outcomes are dichotomised (or otherwise grouped) for analysis. |  |  |
| **12** | **STATISTICAL METHODS: (a) Describe all statistical methods, including those used to control for confounding. (b) Describe any methods used to examine subgroups and interactions. (c) Explain how missing data were addressed. (d) If applicable, describe analytical methods taking account of sampling strategy. (e) Describe any sensitivity analyses.**   - Statistical methods should be clearly described, ideally correlations between scores of cognition and lesion volume. - Unadjusted correlations should be calculated prior to controlling for potential confounders. If either unadjusted correlations or controlling for confounders is not included, a maximum of 0.5 can be awarded. |  |  |
|  | RESULTS | | |
| **13** | **PARTICIPANTS: (a) Report numbers of individuals at each stage of study—e.g. numbers potentially eligible, examined for eligibility, confirmed eligible, included in the study, completing follow-up, and analysed. (b) Give reasons for non-participation at each stage. (c) Consider use of a flow diagram.**   - Participants recruited but not completing either cognitive testing or imaging should be specified. If this is unclear, a score of 0 is awarded. |  |  |
| **14** | **DESCRIPTIVE DATA: (a) Give characteristics of study participants (eg demographic, clinical, social) and information on exposures and potential confounders. (b) Indicate number of participants with missing data for each variable of interest.**   - Summary statistics for basic demographic data (age, sex) and MS phenotype should be provided. If this is not given, a score of 0 will be awarded. - Information on recent steroid use and disease-modifying therapy is considered necessary for a score of 1, but not full results of all potential confounders. Ideally these would be provided in supplementary material. - If results of multiple cognitive tests are used for analysis, the number of participants with incomplete data for each test should be given. If this is unclear, a maximum of 0.5 can be awarded. |  |  |
| **15** | **OUTCOME DATA: Report numbers of outcome events or summary measures.**   - Summary statistics should be presented for both cognitive outcomes and lesion volumes. These should include measures of the dispersion as well as central tendency. Where this is incomplete, e.g. only the numbers of participants categorised as cognitively impaired versus not impaired are provided, a maximum score of 0.5 can be awarded. |  |  |
| **16** | **MAIN RESULTS: (a) Give unadjusted estimates and, if applicable, confounder-adjusted estimates and their precision (eg, 95% confidence interval). Make clear which confounders were adjusted for and why they were included. (b) Report category boundaries when continuous variables were categorized. (c) If relevant, consider translating estimates of relative risk into absolute risk for a meaningful time period.**   - Unadjusted outcomes should be presented for cognitive data and, if applicable, confounder-adjusted outcomes. A measure of their precision should be provided. |  |  |

**Note**

Several papers referred to previous publications for details of cohort recruitment, imaging and analysis methodology. Where necessary for systematic review analysis, this data was sought and used. However the scores awarded as above only cover information presented in the primary publication under review.
