# Supplementary material for: The clinico-radiological paradox of cognitive function and MRI burden of white matter lesions in people with multiple sclerosis: A systematic review and meta-analysis
Source: PLoS One. 2017 May 15;12(5):e0177727. doi: 10.1371/journal.pone.0177727 (PMC5432109; doi:10.1371/journal.pone.0177727)
Supplement: S3 Appendix — (DOCX) [file pone.0177727.s003.docx]

**S3 Appendix:** **Sub-analysis of studies relating T1 hypointense lesion volume to overall cognitive performance.**

Twenty studies included in our systematic review examined the relation of T1 hypointense lesion volume to measures of overall cognitive performance, with 11 providing results which could be converted to a common effect size. Standardised mean differences (seven studies) ranged from -2.16 to -0.14 (equivalent to correlation coefficients of -0.73 to -0.05). Four studies directly calculated correlation coefficients with values ranging from -0.45 to -0.22. Two further studies reported their findings as non-significant. The overall effect size was *r* = -0.26 (95% CI: -0.32,-0.20), see Figure S3a. The total participant number for the 13 studies used in deriving this figure was 1062. There was evidence of heterogeneity (Q = 20.4, df = 10, p = 0.025, *I^2^* = 51.0%). An alternative random effects meta-analysis, using DerSimonian and Laird methodology, gave a summary effect size of *r* = -0.30 (95% CI: -0.39,-0.20). The heterogeneity statistics and random effects meta-analysis were carried out using only the studies providing specific estimates of the effect size (n=11), as per the main analysis.

To investigate the heterogeneity, a funnel plot was drawn (Figure S3b). Egger’s regression test confirmed evidence of funnel plot asymmetry (p = 0.032).

**Figure S3a:** Forest plot of effect sizes from individual studies relating T1 hypointense lesion burden to overall cognitive performance, with 95% confidence interval (total n = 1062). Manuscripts reporting “non-significant” results without a point estimate are represented by circles. Box sizes are inversely proportional to study variance. The overall effect size is *r* = -0.26 (95% CI: -0.32, -0.20).

Figure S3b: Funnel plot of effect sizes, on Fisher’s z scale, against the inverse of standard error (itself inversely related to study size). The vertical dashed line indicates the summary effect on the same scale (z= -0.28).
