# Supplementary material for: The clinico-radiological paradox of cognitive function and MRI burden of white matter lesions in people with multiple sclerosis: A systematic review and meta-analysis
Source: PLoS One. 2017 May 15;12(5):e0177727. doi: 10.1371/journal.pone.0177727 (PMC5432109; doi:10.1371/journal.pone.0177727)
Supplement: S4 Appendix — (DOCX) [file pone.0177727.s004.docx]

**S4 Appendix: Sub-analysis of studies relating T2 hyperintense lesion volume to Symbol Digit Modalities Test (SDMT) performance.**

Thirteen studies included in our systematic review either provided outcome measures relating T2 hyperintense lesion volume to performance in the SDMT (n=11) or specified a non-significant relationship (n=2). Reported correlation coefficients ranged from -0.66 to -0.18 (Figure S4a). The overall effect size was *r* = -0.37 (95% CI: -0.43,-0.31). The total participant number for the 13 studies used in deriving this figure was 885. There was evidence of heterogeneity (Q = 30.7, df = 10, p = 0.001, *I^2^* = 67.4%). An alternative random effects meta-analysis, using DerSimonian and Laird methodology, gave a summary effect size of *r* = -0.45 (95% CI: -0.55,-0.33). The heterogeneity statistics and random effects meta-analysis were carried out using only the studies providing specific estimates of the effect size (n=11), as per the main analysis.

To investigate the heterogeneity, a funnel plot was drawn (Figure S4b). Egger’s regression test confirmed evidence of funnel plot asymmetry (p = 0.0001).

Figure S4a: Forest plot of effect sizes from individual studies relating T2 hyperintense lesion burden to SDMT performance, with 95% confidence interval (total n = 885). Manuscripts reporting “non-significant” results without a point estimate are represented by circles. Box sizes are inversely proportional to study variance. The overall effect size is *r* = -0.37 (95% CI: -0.43, -0.31).

Figure S4b: Funnel plot of effect sizes, on Fisher’s z scale, against the inverse of standard error (itself inversely related to study size). The vertical dashed line indicates the summary effect on the same scale (z= -0.39).
