# Supplementary material for: The clinico-radiological paradox of cognitive function and MRI burden of white matter lesions in people with multiple sclerosis: A systematic review and meta-analysis
Source: PLoS One. 2017 May 15;12(5):e0177727. doi: 10.1371/journal.pone.0177727 (PMC5432109; doi:10.1371/journal.pone.0177727)
Supplement: S5 Appendix — (DOCX) [file pone.0177727.s005.docx]

**S5 Appendix: Sub-analysis of studies relating T2 hyperintense lesion volume to Paced Auditory Serial Additions Test (PASAT) performance.**

Fifteen studies included in our systematic review either provided outcome measures relating T2 hyperintense lesion volume to performance in the PASAT (n=14) or specified a non-significant relationship (n=1). Reported correlation coefficients ranged from -0.58 to -0.07 (Figure S5a). The overall effect size was *r* = -0.28 (95% CI: -0.34,-0.22). The total participant number for the 15 studies used in deriving this figure was 1103. There was evidence of heterogeneity (Q = 29.2, df = 13, p = 0.006, *I^2^* = 55.5%). An alternative random effects meta-analysis, using DerSimonian and Laird methodology, gave a summary effect size of *r* = -0.35 (95% CI: -0.44,-0.26). The heterogeneity statistics and random effects meta-analysis were carried out using only the studies providing specific estimates of the effect size (n=14), as per the main analysis.

To investigate the heterogeneity, a funnel plot was drawn (Figure S5b). Egger’s regression test confirmed evidence of funnel plot asymmetry (p < 0.0001).

Figure S5a: Forest plot of effect sizes from individual studies relating T2 hyperintense lesion burden to PASAT performance, with 95% confidence interval (total n = 1103). A manuscript reporting “non-significant” results without a point estimate is represented by a circle. Box sizes are inversely proportional to study variance. The summary effect size is *r* = -0.28 (95% CI: -0.34, -0.22).

Figure S5b: Funnel plot of effect sizes, on Fisher’s z scale, against the inverse of standard error (itself inversely related to study size). The vertical dashed line indicates the summary effect on the same scale (z= -0.29).
