# Supplementary material for: The clinico-radiological paradox of cognitive function and MRI burden of white matter lesions in people with multiple sclerosis: A systematic review and meta-analysis
Source: PLoS One. 2017 May 15;12(5):e0177727. doi: 10.1371/journal.pone.0177727 (PMC5432109; doi:10.1371/journal.pone.0177727)
Supplement: S8 Appendix — (DOC) [file pone.0177727.s008.doc]

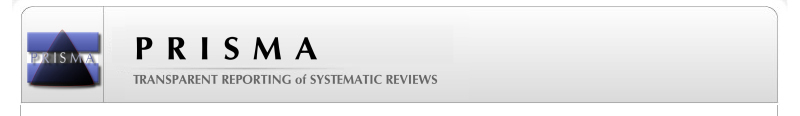
**PRISMA 2009 Flow Diagram – for PONE-D-17-03500:**

**‘The clinico-radiological paradox of cognitive function and MRI burden of white matter lesions in people with multiple sclerosis: a systematic review and meta-analysis.’ [Mollison *et al* (2017)]**

**Screening**

**Included**

**Eligibility**

**Identification**

Records identified through database searching
(n = 3882)

Additional records identified through other sources
(n = 1)

Records after duplicates removed
(n = 1908)

Records screened
(n = 1908 )

Records excluded
(n = 1768)

Full-text articles assessed for eligibility
(n = 139 )

Full-text articles excluded, with reasons
(n = 90)

Aim not relevant to review: 35

No total lesion volume data: 17

CIS/Probable MS subjects: 16

Longitudinal studies: 13

Duplicate subjects/Secondary analysis: 8

Not measuring cognition: 1

Aim not relevant to review: 35

No total lesion volume data: 17

CIS/Probable MS subjects: 16

Longitudinal studies: 13

Duplicate subjects/Secondary analysis: 8

Not measuring cognition: 1

Studies included in qualitative synthesis
(n = 50)

Studies included in quantitative synthesis (meta-analysis)
(n = 32)
