# Supplementary material for: The clinico-radiological paradox of cognitive function and MRI burden of white matter lesions in people with multiple sclerosis: A systematic review and meta-analysis
Source: PLoS One. 2017 May 15;12(5):e0177727. doi: 10.1371/journal.pone.0177727 (PMC5432109; doi:10.1371/journal.pone.0177727)
Supplement: S1 Table — (DOCX) [file pone.0177727.s011.docx]

**S1 Table: Cognitive assessment and scoring protocols**

| Paper | Cognitive tests | Controls | Normative data | Definition of cognitive impairment |
| --- | --- | --- | --- | --- |
| Sacco 2015 | BRB-N, Stroop test | YES | YES (Italian population) | ≥ 2 SD below population mean on ≥ 2 subtests, including one memory test. |
| Yildiz 2014 | ‘MUSIC' battery | NO | NO | Overall score <20/30. |
| Laffon 2014 | PASAT-3s | YES | YES (French population) | ≥ 2 SD below population mean |
| Niino 2014 | BRB-N | YES | Unclear | N/A |
| Sbardella 2013 | PASAT - 2s/3s | NO | YES (Published reference data) | N/A |
| Mike 2013 | 3 ‘Theory of Mind’ tests | YES | NO (Direct comparison with controls) | N/A |
| Francis 2013 | MACFIMS | NO | YES (Published reference data) | >1.5 SD below normative mean on any subtest, and ≥2 abnormal tests |
| Rossi 2012 | BRB-N | NO | YES (Italian population) | ≥2 SD below mean for ≥2 subtests |
| Mesaros 2012 | BRB-N | NO | YES (Published reference data) | ≥2 SD below mean on 1 subtest and ≥1.5 SD on another; or ≥1.5 SD below mean on ≥3 tests. For individual tests ≥1.5 SD below mean. |
| Lund 2012 | 32 tests covering 8 cognitive domains | Unclear | YES (Controls unspecified) | N/A |
| Bomboi 2011 | MACFIMS (without PASAT) | YES | NO | N/A |
| Mike 2011 | MACFIMS | NO | NO | N/A |
| Akbar 2010 | BRB-N (without SDMT) | NO | YES (From published manual) | ≤ 5th percentile on ≥ 2 subtests |
| Heesen 2010 | SDMT for screening; then 6 further tests inc. PASAT | NO | Unclear | Grouped by SDMT: either above mean or ≥1.5 SD below mean |
| Patti 2009 | BRB-N, Stroop | NO | YES (Italian population) | ≥1 SD below mean on ≥3 subtests (N.B. other definitions considered, e.g. ≤ 5th percentile) |
| Krause 2009 | Facial emotion matching test | YES | YES (Controls) | ≥2 SD below control mean |
| Sanchez  2008 | 20+ tests, inc. PASAT, SDMT, Stroop, COWAT, CVLT, SPART, and WCST) | YES | YES (Controls) | Overall cognitive index ≤ 5th percentile of control data |
| Rovaris 2008 | 10+ tests, inc. PASAT and WCST | NO | YES (Italian population) | Scores of 0 on ≥2 subtests (Scoring 0-4 relative to distribution of normative data.) |
| Karlinska 2008 | 5 tests | YES | NO | Unclear |
| Lin 2008 | PASAT (3s) | YES | YES (Published reference data) | ≥2 SD below mean |
| Amato 2008 | BRB-N, Stroop | NO | YES (Italian population) | ≥2 SD below mean on ≥3 subtests |
| Houtchens 2007 | MACFIMS | YES | NO | N/A |
| Parmenter 2007 | WCST & DKEFS | YES | NO | N/A |
| Lazeron 2006 | 8 information processing tasks from computerised battery ‘ANT’ | NO | YES (Normative data) | N/A |
| Benedict 2006 | PASAT, SDMT, BVMT-R, CVLT-II | YES | YES (Previously published control data) | ≥2 SD below mean on 1 test and ≥1.5 SD on another, or ≥1.5 SD below mean on ≥3 tests. |
| Lazeron 2005 | BRB-N (+/- substitution for SRT) | NO | YES (Published reference data) | Overall scores ≤2 SD below reference mean |
| Deloire 2005 | BRB-N + 5 tests, inc. Stroop | YES | YES (Controls) | ≤ 5th percentile of control data |
| Archibald 2004 | 2 information processing tasks | NO | YES (Previously published control data) | N/A |
| Benedict 2004 | MACFIMS (without DKEFS), WCST and one other | YES | NO | N/A |
| Christodoulou 2003 | BRB-N + 1 other | NO | YES (published data and previous study subjects) | N/A |
| Bermel 2002 | SDMT | NO | NO | N/A |
| Zivadinov 2001 | 9 tests, inc. PASAT, Stroop | YES | YES (Partly published cut-offs, partly control data) | Abnormal result on ≥2 tests: ≤2 SD below control mean or published cut-off |
| Nocentini 2001 | 20 tests, inc. SDMT, Stroop | YES | NO | U |
| Kalkers 2001 | PASAT-3s | NO | YES (Published reference population, partly overlapping) | N/A |
| Snyder 2001 | PASAT (4 speeds) | NO | NO | N/A |
| Comi 1999 | Frontal battery, inc. WCST, Stroop | NO | YES (Italian population data or previous controls) | Published cut-offs or ≤5^th^/10^th^ percentile of controls. Overall groups: either ≥3 abnormal subtests, or normal in all. |
| Camp 1999 | BRB-N, plus one other. | YES | YES (Controls) | N/A |
| Sun 1998 | 2 dementia screening scales, inc. MMSE | NO | NO | N/A |
| Rovaris 1998 | 10 tests, inc. PASAT, SRT, SPART, WCST, Stroop | NO | NO | U: Abnormal results in ≥2 tests |
| Hohol 1997 | BRB-N | NO | NO (Published guidelines) | N/A |
| Patti 1995 | 2 dementia screening scales, plus 2 other tests | NO | NO | U |
| Comi 1995 | 10+ tests, inc. WCST, JLO. | NO | YES (Italian population) | ≥3 subtests ≥2 SD below mean |
| Moller 1994 | SIDAM dementia screening interview, inc. MMSE | NO | NO | ≤46/55 |
| Swirsky-Sacchetti 1992 | 8 tests, inc. CVLT, COWAT,WCST | NO | YES (Partly, published normative data) | N/A |
| Ron 1991 | 10+ subtests, inc. WCST | YES | YES (Controls) | N/A |
| Pozzilli 1991 | 10 tests, inc. WCST | YES | YES (Controls) | ≥2 SD below control mean on ≥2 subtests |
| Izquierdo 1991 | 5+ tests, inc. MMSE | Unclear | NO | N/A |
| Anzola 1990 | 9 tests, inc. JLO | NO | YES (Published norms) | N/A |
| Franklin 1988 | 10+ tests, inc. SDMT | YES | YES (Previous standardisation sample) | ≤16^th^ percentile of normative data |
| Huber 1987 | 8 test, inc. MMSE | YES | NO | ≥1,2 or 3 SD below control mean |

**S1 Table:** Summarising cognitive testing and scoring protocols, with commonly used tests and batteries named.

*Abbreviations:* BRB-N: Rao’s brief repeatable battery, including selective reminding test (SRT), spatial recall test (SPART), SDMT, PASAT, controlled oral word association test (COWAT); MACFIMS: Minimal Assessment of Cognition in Multiple Sclerosis, including PASAT, SDMT, COWAT, California verbal learning test (CVLT-II), Brief visuospatial memory test (BVMT-R), Delis-Kaplan executive function system (D-KEFS), judgment of line orientation (JLO). Stroop: Stroop colour-word matching test. MMSE: Mini Mental State Examination. N/A: not applicable.
